# Chicken Secondary Lymphoid Tissues—Structure and Relevance in Immunological Research

**DOI:** 10.3390/ani14162439

**Published:** 2024-08-22

**Authors:** Cassandra Ceccopieri, Jan P. Madej

**Affiliations:** Department of Immunology, Pathophysiology and Veterinary Preventive Medicine, Wroclaw University of Environmental and Life Sciences, 50-375 Wroclaw, Poland; cassandra.ceccopieri@upwr.edu.pl

**Keywords:** poultry, secondary lymphoid organ, interspecies similarity, animal model, MALT

## Abstract

**Simple Summary:**

Birds are highly exposed to pathogens leading to high risk of low egg production, high mortality rates and high costs of veterinary care. Moreover, the intense breeding of avian species for desirable production traits causes immunosuppressive stress. Therefore, line selection requires thorough knowledge not only of genetics but also of the immunology of poultry. The function of secondary (peripheral) lymphoid organs is to activate the immune response through the accumulation of antigens for the activation of naive B and T cells. This review aims to present the morphological characterization of chickens’ secondary immune organs along with the latest and more outstanding scientific discoveries and their relevance not only in veterinary medicine but also in human medicine. The authors hope that this review will represent a useful source of information for the design of future experiments with animal models in terms of more tailored protocols and heightened awareness.

**Abstract:**

Recent discoveries have indicated the importance of developing modern strategies for vaccinations, more ethical research models, and effective alternatives to antibiotic treatment in farm animals. Chickens (*Gallus gallus*) play a crucial role in this context given the commercial and economic relevance of poultry production worldwide and the search for analogies between the immune systems of humans and birds. Specifically, chicken secondary lymphoid tissues share similar features to their human counterparts. Chickens have several secondary or peripheral lymphoid tissues that are the sites where the adaptive immune response is initiated. The more general classification of these organs divides them into the spleen and skin-, pineal-, or mucosa-associated lymphoid tissues. Each of these tissues is further subdivided into separate lymphoid structures that perform specific and different functions along the animal’s body. A review summarizing the state of the art of research on chicken secondary lymphoid organs is of great relevance for the design of future studies.

## 1. Introduction

**Figure 1:**
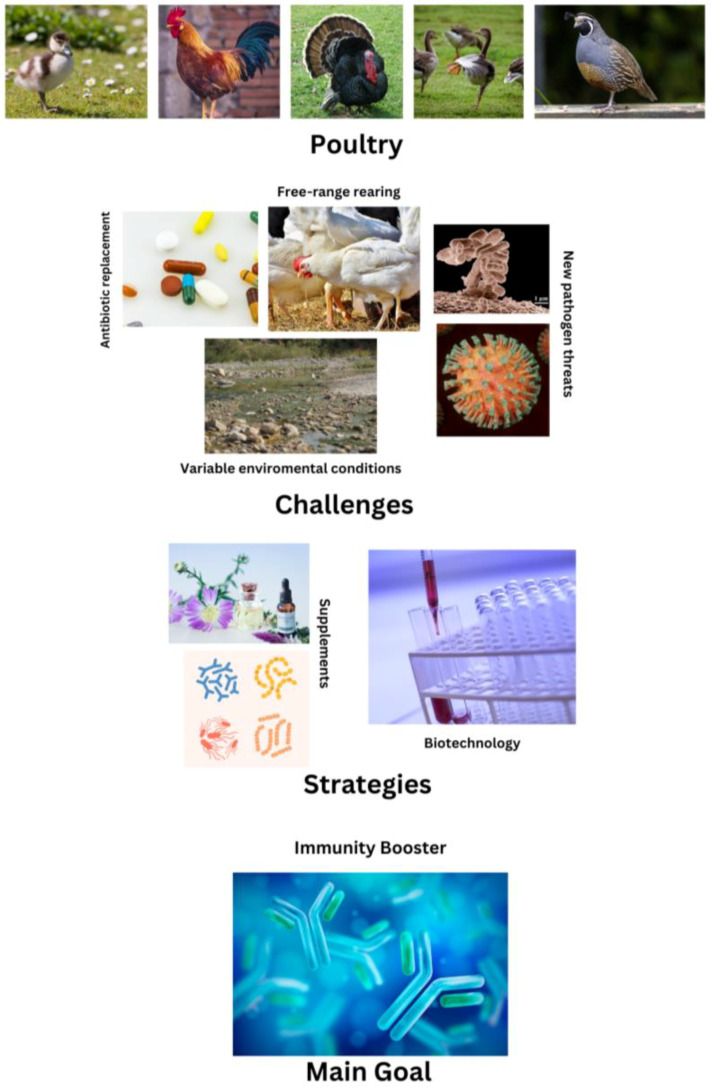
Summary of the current challenges in poultry science, highlighting the pivotal role of immunological research as a key to solving the present issues.

Avian immunology is experiencing a prolific era of research given the increasing interest in vaccine design [1,2] and the development of more sustainable and ethical models employing chicken embryos [3]. Furthermore, avian infectious diseases caused by new strains of existing pathogens [4] or new infective agents [5,6] cause great economic losses every year. Indeed, this entails decreased egg production, higher mortality rates, and higher costs in veterinary care [7]. Environmental changes, variations in legislation or market forces, pathogen evolution, and the establishment of large-scale production continuously transform the nature of the infectious diseases threatening poultry. Moreover, the intense breeding of avian species for desirable production traits causes immunosuppressive stress [8]. The increasing interest in animal welfare is driving a move to outdoor rearing, which results in changes to the spectrum of pathogen exposition [9]. Free-range rearing increases, even more, the threat of pathogen spillover at the agriculture–wildlife interface [10]. Therefore, the development of poultry lines selected for disease resistance and improved immunity becomes more and more important [11,12,13]. Line selection requires a thorough knowledge not only of genetics but also of the immunology of poultry [11,14] (Figure 1).

The immunology dogma defines the immune responses as divided into two branches: humoral and cell-mediated [3,15]. The humoral responses are carried out by soluble indicators, including acute-phase proteins, cytokines, and antibodies that can be found in blood and other tissue fluids [16]. Cell-mediated responses primarily consist of clearing effectors, such as cytotoxic T cells, macrophages, and natural killers (NKs), that are able to lyse or phagocyte pathogens or infected cells. Humoral and cellular immunity are constantly entangled. Once antigen-presenting cells (APCs) activate T helper cells and trigger the release of cytokines, a series of reactions follows that is aimed at neutralization of the infective agents [17].

The tissues and organs of the immune system can be classified as either primary, in which lymphocytes are generated and undergo development and maturation, or secondary, where mature lymphocytes interact with antigens. In chickens, the primary lymphoid organs are the thymus, where T lymphocytes originate, mature, and proliferate, and the bursa of Fabricius, where B lymphocytes undergo similar processes [18].

Secondary or peripheral lymphoid organs are the sites of the activation of the immune response following interaction with a pathogen [19]. They exert a modulation of the immune reaction that shifts between cell-mediated and humoral responses through a nonlinear process highly dependent on the kind of source antigen. Based on the variable nature of the antigen presented by the APC to the naive CD4^+^ T cell, different interleukins (ILs) will be released, leading to differentiation between the T cells in different subclasses [20]. While the T helper 1 (Th1) subclass releasing IL-2 and IFNγ is involved in the activation of cellular-mediated responses, the T helper 2 (Th2) subclass mediates the activation of humoral responses through the release of IL-4, 5, and 13 and the activation of B cells [21].

In chickens, secondary immune organs consist of the spleen and conjunctiva-, gonad-, mucosa-, pineal-, and skin-associated lymphoid tissues. The spleen, the largest peripheral lymphoid structure, is a defined organ located on the dorsal left side of the proventriculus [22] and it is the primary site for immune responses against blood-derived antigens [23]. Mucosa-associated lymphoid tissue (MALT) can be found scattered throughout the body [24]. The lymphoid structures of MALT are indeed present in the digestive system, the eyes (Harderian glands) [25], the skin, and the respiratory system [26].

The aim of this review is to collect information on the histological characterization of chickens’ secondary immune organs, along with information on the latest and more outstanding scientific discoveries related to each of the structures presented in this work and their relevance not only in veterinary medicine but also in human medicine.

The authors hope that this review represents a useful compendium of information for the design of future experiments in terms of achieving more tailored protocols and a more-aware use of animal models.

## 2. Secondary Lymphoid Organs

### 2.1. Spleen

Morphologically and functionally, the chicken spleen is composed of two sections: white pulp and red pulp. While in the mammalian spleen there is a defined marginal zone of separation between these two areas, in chickens, this border is made up of a complex ellipsoidal sheath of reticular cells and a layer of B cells surrounded by a ring of macrophages [23]. Chickens’ red pulp differs considerably from its mammalian counterpart, mainly in the system of blood circulation within the organ [22]. In chickens, there is a closed circulation system with capillaries entering the red pulp and then directly entering the sinuses, while in mammals, this system is open, with penicillar capillaries opening into the pulp cords from where the blood enters the sinuses [22]. The chicken spleen includes three lymphoid regions: the lymphoid tissue of the T lymphocytes surrounding the central arteries, referred to as the periarteriolar lymphoid sheath (PALS); the lymphoid tissue of the B lymphocytes surrounding the branching penicillary capillaries, which takes the name of peri-ellipsoidal white pulp (PWP); and the germinal centers where B lymphocytes proliferate and differentiate [23]. The red pulp is made up of a 3D meshwork of spleen sinuses and cords and is responsible for blood filtering, i.a., from the circulating pathogens [23]. Lymphocytes, hematopoietic cells, and plasma cells are associated with the splenic cords along with the plasmablasts that migrate from the follicles and the outer PALS. The macrophages located in the red pulp exhibit active phagocyte activity, enabling the removal of senescent or damaged erythrocytes and blood-derived particulate matter [23].

**Figure 2:**
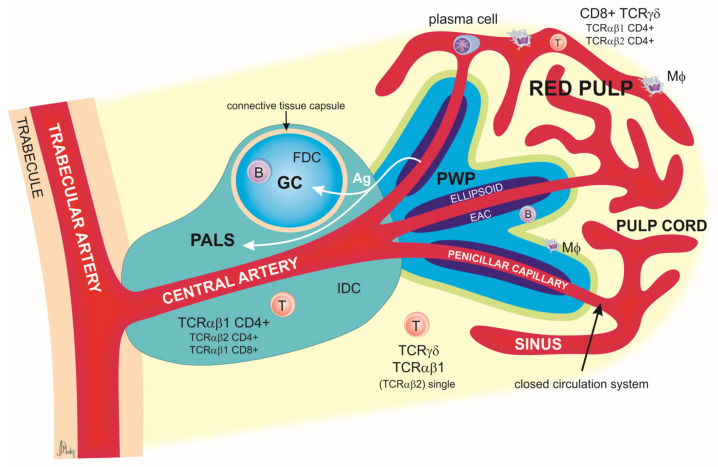
Schematic representation of splenic lobules in chicken. The lineal artery enters the organ and then divides into the trabecular arteries, which, in turn, branch out and enter the pulp as central arteries. The latter divide into smaller central arterioles and finally into the penicillar capillaries associated with venous sinuses. Central arteries are surrounded by a periarteriolar lymphoid sheath (PALS). The PALS is divided into the inner PALS and the outer PALS. The inner PALS contains mainly T lymphocytes expressing CD4 and TCRαβ1 molecules. TCRαβ2^+^ CD4^+^ and TCRαβ1^+^ CD8^+^ cells are also present. Between the T cells are located interdigitating dendritic cells (IDCs), most likely the precursor of follicular dendritic cells (FDCs). In the outer PALS are small and medium lymphocytes (both B cells and T cells), macrophages (MΦ), and plasma cells after activation. At the border of the trabecular and central arteries are located the germinal centers (GCs), which are composed primarily of B cells but in which a small number of follicular dendritic cells and CD4^+^ T cells can also be found.

The main structure and histological organization of the chicken spleen were described in great detail in 1991 by Jeurissen [22], as summarized in Figure 2.

#### Relevance of the Spleen in Immunological Research

The current research involving the chicken spleen is focused on several aspects. Of great interest is the characterization of the precise typology and immunological functions of innate immune cells of the organ. For instance, Sutton et al. [27] uncovered a pattern of localization of conventional dendritic cells and macrophages in the chicken spleen. According to this study, in the areas surrounding the PALS, there is a prevalence of macrophages expressing mannose receptor C1-like B (MRC1L-B), while in the peri-ellipsoidal white pulp, there is a high expression of putative CD11c^+^ macrophages. Furthermore, these authors discovered a new subset of macrophages called “ellipsoid macrophages”, which express MHCII, CD11c, MRC1L-B, and the receptor for colony-stimulating factor 1 (CSF1R) [27]. These results updated the classification of chicken mononuclear macrophages that previously considered MCR-1 as a marker for cells of monocyte/macrophage lineage and CD11 as a marker for a dendritic-like cells. Furthermore, their study contributed greatly to the annotation of the mononuclear phagocyte system receptors’ repertoire that is crucial for antigen uptake and presentation processes.

Another area of interest concerning the chicken spleen touches on the process involved in lymphocyte homing. Some researchers have reported that, following stimulation with lipopolysaccharides, upregulation of integrin β1 and V-CAM-1 in the spleen endothelial cells was observed [28]. This activation of the endothelium allows the migration of the lymphocytes to the spleen’s red pulp [28]. This represents a fundamental process on which depends the efficacy of the whole immune system. Lymphocyte homing indeed not only controls lymphocytes’ differentiation and survival but is also responsible for targeting immune effector cells to the site of contact with antigens [28].

The chicken spleen (along with the liver) has also been used as a model organ to evaluate the profile of active tissue kinases [29]. This project was of great importance for improving the knowledge of the regulatory pathways controlling chickens’ physiological processes. This class of enzymes covers indeed a pivotal role in cellular activation processes, including immune receptors’ signal transduction [30]. Furthermore, the characterization of a repertoire of catalytically active kinases represents an invaluable asset in drug design and inhibitor selectivity studies [29,31]. One of these studies uncovered that, under the conditions of sample collection, the number of catalytically active kinases was higher in the spleen than in liver specimens. Among those active enzymes, three kinases were found to be involved in essential cell pathways: IKKa, AKT1, and PIK3CA [29]. These results contribute to the annotation of the chicken kinome and provide insights into the role of kinases in chicken development, growth, and pathophysiology. Advances in the knowledge of kinases’ basic science in any model species allow for the development of new therapeutic approaches with revolutionary outcomes in human medicine.

Over the last two decades, poultry research has been intensively focused on uncovering the molecular regulatory mechanism of stress reaction given its implication in productive performance [32]. In 2020, a study was published containing a list of immune-related genes susceptible to stress in the Gushi cock, a Chinese local breed of chicken [33]. Several of the stress-sensitive genes identified during this study are involved in immunological functions like T cell-mediated immunity and chemokine-mediated signaling pathways. Further studies revealed that heat stress, in particular, leads to impairment of the development and functional maturation of T and B cells in primary and secondary lymphoid tissues of broiler chickens [34]. The dynamics of gene expression in chicken spleen under corticosterone-induced stress conditions have been described in detail by Su et al. [35]. Their study revealed that a high percentage of the genes differentially expressed under stressed conditions were involved in immune functions, such as cytokine–cytokine receptor interactions, JAK-STAT signaling pathway mechanisms, and RLR signaling mechanisms [35].

### 2.2. Mucosa-Associated Lymphoid Tissue (MALT)

Chickens, unlike mammals, lack encapsulated lymph nodes but instead develop diffuse lymphoid tissue generating in the sites of antigenic stimulation. This feature represents the most striking distinction between these two models of the immune system [3]. A great example of diffuse lymphoid tissue is represented by the MALT, which consists mainly of immune cells such as dendritic cells and T and B lymphocytes [36]. Upon stimulation by luminal antigens, the mucosal immune cells infiltrate into diffuse areas of the mucosa (e.g., the respiratory mucosa and lamina propria of intestinal villi) and carry out immune effector functions [2].

The mucosa represents an extensive physical barrier preventing pathogen invasion. In birds, its structure is extremely complex, suggesting a wider spectrum of functions compared with its mammalian counterpart [2]. MALT assists with the development of longer-lasting local immunity, mainly in the respiratory tract but also in the gastrointestinal mucosal linings and the oviduct as part of genital organ-associated lymphoid tissue (GOALT) [37].

#### 2.2.1. Nasal-Associated Lymphoid Tissue (NALT)

The nasal mucosa is the first site of interaction with airborne contaminants. Along the respiratory tract, numerous clusters of lymphoid tissue are present, namely conjunctiva-associated lymphoid tissue and paranasal glands that altogether make up the NALT [38]. The major structures found in chickens’ NALT are lymphoid nodules covered by a nonciliated epithelium, predominantly containing B cells organized in germinal centers and surrounded by a layer of CD4^+^ T lymphocytes. Most B cells are IgY^+^, while IgA^+^ and IgM^+^ are scarce [38]. In the orbit of the eye is then present another important secondary lymphoid organ called the Harderian gland, rich in B cells and plasma cells organized in germinal centers, and macrophages and scattered T cells organized in T cell-dependent interfollicular regions. The Harderian gland is responsible for adaptive responses upon ocular exposure to pathogens [38].

#### 2.2.2. Tracheal Mucosal Immunity

Following the structure of the respiratory tract, the trachea is then found, where, throughout the mucosa, scattered lymphoid cells can observed, mainly mononuclear phagocytes. These cells make the tracheal mucosa highly responsive to infections with extensive lymphocyte infiltration and lymphoproliferation [39].

#### 2.2.3. Bronchus-Associated Lymphoid Tissue (BALT)

The bronchus-associated lymphoid tissue mediates the bronchial immunity. It consists of intrapulmonary lymphoid tissue connected to the pulmonary vessels and adventitia of the bronchi [40,41]. In the nodules of the mature BALT, there are germinal centers surrounded by CD4^+^ T cells. The CD8^+^ lymphocytes are mainly found dispersed between nodules and in the epithelium. T and B cells colonize the bronchi at around two weeks after hatching with the production of an equal number of IgY^+^, IgA^+^, and IgM^+^. The proportion changes soon after in favor of IgY^+^ and IgM^+^ [40].

#### 2.2.4. Gut-Associated Lymphoid Tissue (GALT)

Another important example of MALT is represented by the GALT. The epithelium of the intestine represents an extensive physical barrier that selectively allows the absorption of nutrients and the excretion of waste while preventing pathogen invasion [42]. GALT is able to be paracellularly permeated through different types of intercellular junctions, representing a great strategy against microbial invasion. The abundance of different typologies of tight junctions is decisive for characterizing the different levels of the epithelium’s permeability throughout the intestinal tract [43]. The structure of the lymphoid tissue is finely organized in an interfollicular space rich in T cells, and follicles with germinal centers rich in B cells [43]. In the lamina propria, one can find T cells expressing CD4 and TCRαβ1 and B cells. Throughout the lamina propria are then scattered CD8^+^ TCRγδ cells and CD4^+^ TCRαβ2 cells, while in the villi, IgM^+^, IgA^+^, and IgY^+^ plasma cells are found [43].

##### Esophageal Tonsils

The esophageal tonsil is a lymphoid structure located at the junction of the esophagus and proventriculus. The main gut-associated lymphoid organs are located at the anatomical junctions of different parts of the gastrointestinal tract (GIT), always caudal to the stomach [44]. On the contrary, esophageal tonsils are situated cranial to the stomach; therefore, they are exposed to undigested food, infectious agents, and other antigens. The esophageal tonsils’ lymphoid tissue is divided into two parts: germinal centers or follicles and interfollicular lymphoid tissue, which, respectively, form the B- and T-dependent areas. The esophageal tonsils are isolated from the environment by a thin layer of fibrous connective tissue called the capsule [44]. Lymphoid cells infiltrate the stratified squamous epithelium of the esophagus, creating the lymph-epithelium that is present mainly in the crypts of the tonsillar unit and in the excretory duct of each of the esophageal glands. Major Histocompatibility Complex (MHC) class II positive stellate cells and dendritic or dendritic-like cells can be found in the lymph-epithelium of the esophageal tonsils along with plasma cells able to migrate from the tunica propria into the epithelium. The germinal centers of the tonsils are sharply outlined by a connective capsule and can have highly variable sizes and shapes. B lymphocytes are mainly located in the follicles, while T cells are mainly located in the interfollicular areas [44].

##### Pyloric Tonsils

Lymphoepithelial structures called pyloric tonsils are found as complete lymphoid ring occupying the entire wall of the GIT at the beginning of the duodenum [45]. Like the esophageal ones, pyloric tonsils are also characteristic of the chicken and absent in mammals. These structures are composed of at least 15–20 tonsillar units with a sharp delineation in both proximal and distal directions. Each tonsillar unit is indeed surrounded by a capsule with a high content of collagen type III. The lymphatic cells present in the interfollicular region of these tonsillar units are mainly CD45-positive hemopoietic cells (found also in the germinal centers) and CD3^+^ T cells. On the contrary, B cells in pyloric tonsils are restricted to GCs, present in a remarkably high number [45].

##### Meckel’s Diverticulum (MD)

Meckel’s diverticulum, an anatomical landmark in birds, consists of an appendage of the small intestine made of the remnant of the yolk stalk [46]. After hatching, a great portion of the yolk is translocated into the intestine to provide a food supply for the chick [36]. In the lymphopoietic tissue of MD, three zones are distinguished based on the cellular content. Monocytes are in the zone closest to the lumen of the yolk sac, undifferentiated blast cells are found in the middle zone, and immature granulocytic cells populate the largest external zone [47]. Mononuclear phagocytes are dispersed over the MD as single cells. With age progression, the lymphoid tissue gradually expands and fills the folds. Simultaneously, the number of goblet cells increases, clusters of lymphoblasts are formed inside the folds, and the epithelium is infiltrated by lymphocytes. In a mature chick, separate B and T areas can be distinguished: B cells are located in germinal centers and beneath the epithelium, while T cells are found between germinal centers [47].

##### Peyer’s Patches (PPs)

Peyer’s patches are lymphoid structures present along the alimentary tract consisting of aggregated lymphoid nodules acting as lymphoid inductive sites [48]. The immune cells present in PPs are macrophages, dendritic cells, plasma cells, and B and T cells. Therefore, antigenic stimulation in the PPs can trigger effective mucosal and systemic immune responses [48]. Furthermore, the production of antigen-specific secretory immunoglobulin A (sIgA) can be activated in situ, creating a protective barrier against infection and the invasion of enteric pathogens [48]. PPs are characterized by a follicular structure with thickened villi and a specialized epithelium containing M cells. In the subepithelial zone, B cells predominate. The T cells of the interfollicular zone express TCRαβ1 and are mainly CD4^+^, with only a small percentage of TCRγδ^+^ cells (5%) [49].

##### Cecal Tonsils (CTs)

**Figure 3:**
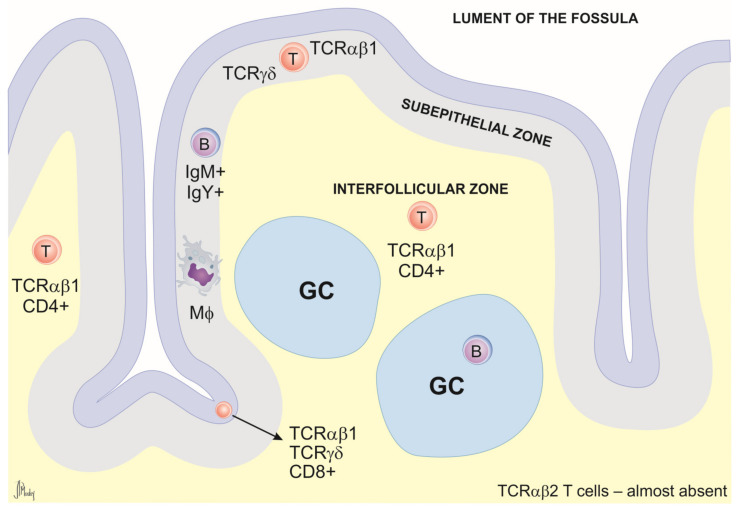
Schematic representation of cecal tonsil cross-section. The general structure includes the epithelium, subepithelial zone, germinal centers (GCs), and interfollicular zones. The B cells are found mainly in GCs and in the subepithelial zone. The interfollicular (T cell-dependent) zone consists mainly of TCRαβ1^+^ CD4^+^ T cells. CD8α^+^ cells are generally present in the epithelium and are scattered through the lamina propria. TCRγδ^+^ cells are usually found in the epithelium and in the subepithelial zone. Macrophages (MΦ) occur scattered through the lamina propria but are most prevalent directly under the epithelium.

Distributed along the entire structure of the galliform ceca, throughout the mucous membrane, are lymphoid nodules that regulate the continuous proliferation of the cecal microflora [50]. Most nodules seem to distribute in the mesenteric mucosa, in particular at the proximal and distal portions of the ceca. Among the cecal lymphoid structures, the main interest of the scientific community remains focused on the cecal tonsils. CTs are indeed the largest lymphoid aggregates of avian GALT responsible for eliciting protective immune responses against bacterial and viral pathogens [51]. They are located at the proximal end of each cecal pouch, on the inner facing wall of the ceca at ileo–cecal junctions [36]. The T cell population includes mainly CD4^+^ and CD8^+^ cells expressing TCRγδ or TCRαβ. B cells are mainly organized in germinal centers but are also found dispersed throughout the lamina propria [52] (Figure 3).

#### 2.2.5. Relevance of MALT in Immunological Research

MALT has been an object of great interest to the pharmacological industries—both the veterinary one and the human one—over the last few decades. Indeed, considering that most infectious diseases begin in mucosal tissues, it is crucial to develop vaccines that can induce immune responses in the mucosa [1,2,53,54,55,56]. The success of the oral vaccination campaign against polio in humans [53] has justified the efforts spent in this direction. For instance, it was proven that, in chickens, following contact with virulent or vaccine strains of infectious bronchitis virus (IBV), MALT aids in the development of longer-lasting local immunity [55]. This happens mainly in the respiratory tract but also along the gastrointestinal mucosal linings and the oviduct [55]. In poultry science, one of the most interesting applications of mucosal vaccination is that against the influenza virus, a highly infectious respiratory pathogen affecting various animal species with high rates of zoonotic transmission and pandemic potential [57,58]. Humans and chickens appear to be capable of building comparable immune responses in response to the same pathogen [3]. Therefore, if a vaccination strategy proves to be effective in chickens, it may also be considered for human application. The complexity of the mucosal barrier has limited the progress of mucosal vaccines (to date, less than ten mucosal vaccines have been approved) [59,60]. However, research efforts have continued, and, at present, there are several ongoing trials for mucosal vaccines against coronavirus disease 2019 (COVID-19) [61,62,63,64], respiratory syncytial virus [65], influenza viruses [66,67,68], HIV [69,70], and some forms of cancer [71].

The development of alternative strategies to improve animal health and welfare in commercial poultry production is also of great interest currently. Reducing the use of antibiotics in farm animals has become a priority. Antibiotic resistance is indeed a threat to not only for animals but also humans. Here is where the gut microbiota comes into play. The control of the gut microbiome has a beneficial effect on immunity performance due to, among other effects, its contribution to physical barriers’ integrity (i.e., mucin secretion), IgA secretion [72], and the regulation of proper immune system development in other organs such as the lung, as explained by the concept of the “gut–lung axis” [73]. In the context of poultry, the advantages of dietary supplementation are exhaustively explained in the review by Al-Khalaifah [74]. The replacement of antibiotics with commensal bacteria and supplements leads to enhanced growth among animals and positive modulation of their immune responses. In ovo administration of commensal bacteria and evaluations of its effect on immunity performance have recently become trending areas of research, with promising results [75,76,77,78,79]. Of great interest is the study of the modulatory effect on lymphoid organs’ development through the regulation of lymphocyte subset proliferation. For instance, the in ovo administration of commensal bacteria influences post-hatching GALT development in chickens, measured as the colonization rate of B and T cells [80]. Recent research by Szczypka et al. [81] showed how in ovo administration of synbiotic inulin and *Lactococcus lactis* subsp. *lactis* positively modulates the immune performance of broiler chickens through an increase in serum IgG levels post immunization.

Another innovative strategy for antibiotic replacement involves the implementation of bacteriophages targeting specific pathogens without disturbing the microbiota [82]. This approach is producing encouraging results in the treatment of salmonellosis, one of the most important zoonotic foodborne pathogens [83].

Herbal extracts and derivatives are also a valid alternative to antibiotics, as extensively described in the reviews by Kuralkar et al. [84] and Paradowska et al. [85]. In broilers, promising results have arisen from the administration of several plant-derived substances, as listed in Table 1.

In the case of layer lines, antimicrobial strategies are more directed towards reducing—or, better, eliminating—the contamination of eggshells [98]. Several natural compounds have been used in this process as an alternative to common disinfectants, including bacteria-derived compounds [99] and plant extracts [100].

Genetic engineering is one of the ultimate strategies to improve animals’ resistance to pathogens [101]. Research studies on chickens’ immunoglobulin genes have led to a better understanding of B cell development and antibody production in this species [11]. Thanks to the advancements in gene-editing techniques (i.e., the CRISPR/Cas9 system), these results have allowed the generation of genetically modified chickens carrying resistance to specific pathogens [101]. A selected mutation in the gene encoding the receptor for avian leukosis virus subgroup J (ALV-J) has been proven to guarantee resistance to highly pathogenic ALV-J infection [12].

The research on viral pathogenesis is constantly being updated, especially in poultry science, given the high susceptibility of these animals to viral infections and the economic impact of virus outbreaks in commercial lines. Chickens’ immune organs are an invaluable system for the evaluation of viruses’ impact on organisms. New results describing molecular pathways related to viral pathogenesis are incoming daily. Of great importance has been the uncovering of differential antiviral profiles in their immune organs. A good example of this is given by the infectious bursal disease virus (IBDV): recently, researchers discovered a diverse pattern of modulation in innate antiviral mechanisms of defense within the intestinal lamina propria of IBDV-infected chicken [58].

### 2.3. Gonad-Associated Lymphoid Tissue (GOALT)

In the chicken oviduct, lymphocytes are present in both scattered and organized forms in nodular aggregates of the mucosa [102]. The intraepithelial lymphocytes are responsible for the first line of the host’s defense against foreign antigens, including various pathogens [103,104]. Cell-mediated immune responses, mainly those mediated by T cells, play an essential role in protecting the host against intracellular pathogens [102].

Ovarian sex steroids play an important role in the modulation of the immune response, with immune-enhancing activities mediated by estrogen and immunosuppressive activities mediated by progesterone. More specifically, it was shown that estrogen enhances the migration of T cells from lymphoid organs to the oviduct, while progesterone inhibits it [105]. Furthermore, the lymphoid tissue and, in particular, the plasma cells of the oviduct might be involved in the transmission of immunity from mother to offspring [106]. The importance of local immunity for reproductive functions of the hens’ gonads was thoroughly described in the review by Yoshimura [107]. A positive effect is exerted by macrophages, T lymphocytes, and NKs. These cells play a crucial role in the removal of cell fragments of atretic follicles, keeping the ovarian microenvironment optimal for reproduction. On the other hand, local immunity may also have a detrimental effect on reproduction with the release of autoantibodies [107]. The ovarian tissue of hens is subjected to many pathogenic agents that can be transmitted through the eggs, causing salpingitis, reduced egg production, or poor eggshell quality in mature chickens, as well as hypoplasia of the ovarian epithelium in immature chicks [107]. The inflammation caused by infection onset interferes with the egg formation process [108] and leads to contamination of the eggs [109]. For instance, IBV alters the gene expression of collagen type I in the isthmus, affecting eggshell integrity [110].

#### Relevance of GOALT in Immunological Research

The current interest is focused on enhancing the mucosal barrier function of the hen’s oviduct [111]. Several strategies have been designed for this, including oral administration of commensal bacteria that can be translocated from the intestine to the oviduct through the cloaca [112]. Recently, intravaginal administration of lactic acid bacteria (i.e., *Lactobacillus johnsonii*) has been used to enrich the microflora and strengthen the mechanical barrier mediated by the tight junctions of the oviductal mucosa, producing promising results [113].

### 2.4. Pineal Gland-Associated Lymphoid Tissue (PALT)

In birds, the pineal gland works as an autonomous circadian pacemaker [9]. More specifically, in chickens, it contains a circadian oscillator and receptors for various physical and biochemical synchronization stimuli. This allows the gland to receive external photic cues and translate them into the rhythmical synthesis of melatonin, a biochemical signal of darkness [114]. When a chicken is around 3 weeks old, its pineal gland begins to be infiltrated by lymphoid cells. This process causes an increase of 50% in the pineal mass and activates the production of antibodies [115]. Interactions between the neuroendocrine system and immune system suggest the presence of feedback mechanisms mediated by immune soluble factors. Microglia/macrophages, through the secretion of cytokines and chemokines, are involved in the mediation of these signals between lymphocytes and pinealocytes [115]. In vitro studies have shown that these cells can regulate pinealocyte neurite length and serotonin content. Moreover, they guide the upregulation of the expression of cytokines, MHC class II, and other surface antigens in response to immune mediators and bacterial wall components [115]. Immunohistochemical phenotyping of PALT’s lymphocytes revealed the presence of a majority of CD3^+^ and CD4^+^ T cells in comparison to B lymphocytes. The chicken pineal gland’s pivotal role in immunity has been described in a detailed review by Markowska et al. [9]. The key to the interplay between pineal gland reactivity and immunity is the pineal hormone melatonin, responsible for the regulation of the rhythmicity of immune reactions during seasonal changes and innate immunity in general [114].

#### Relevance of PALT in Immunological Research

The current research challenges involving chicken pineal glands focus on the effect of different light regimens on poultry performances, including immunity. The relationship between the perception of light and poultry behavior and welfare requires further investigation to be fully elucidated, but several studies have demonstrated its importance. Indeed, the relevance of the lighting regimen is primary in commercial poultry, as is well explained in the review by Wu et al. [116]. Green and blue LED light employment has been found to lead to better performance in broiler chickens, with increased body weight, improved motility, reduced stress responses, and improved immunity compared to animals reared under white light [117]. More recent tests on UV light [118,119] and intermittent short-photoperiod exposition [120] have also shown promising results in relation to overall welfare, fear response, immune efficiency, and stress susceptibility. Currently, one of the biggest challenges remains to be the evaluation of the impact of light regimens on meat quality [116].

### 2.5. Skin-Associated Lymphoid Tissue

The skin, including the feather pulp, is an important part of the avian immune system, carrying specialized immune cells like the Langerhans cells in the epidermis. Several avian viruses like Marek’s disease [121], avian leukosis [122], and chicken infectious anemia virus [123] replicate in the feather pulp and/or the epithelium feather follicle.

### 2.6. Mural Lymph Nodes (MLNs)

Avian mural lymph nodes are lymphoid accumulations (LAs), with limited capacity for filtration, located along the posterior tibial–popliteal and lower femoral veins [124]. MLNs are found in all birds; however, in domestic species, the pattern of localization is characteristic, typically being a few millimeters to several centimeters apart [124,125]. The surface of LAs is covered by a thin capsule of connective tissue enclosing lymphoid and adipose tissues. Afferent and efferent lymphatic vessels are located only on one side of the LA. LAs contain, in their apical section, valves regulating the lymph flow’s direction [125]. A characteristic of LAs is the presence of plasma cells that are not found in the germinal centers of other lymphoid organs. LAs differ from “true” lymph nodes for several anatomical and histological reasons. Firstly, they are associated with the lateral side of the lymphatic system, and hence do not interrupt the lymph flow as mammal lymph nodes do [125]. Secondly, while “true” lymph nodes have reticular fibers and macrophages contributing to the mechanical and biological filtering of afferent lymph, LAs are characterized by the absence of reticular fibers and macrophages in lymph sinuses [125]. Mural LAs can be classified into three different groups representing different developmental stages. The smallest LA type consists of lymphoid infiltrations surrounded by adipose cells in the wall of the lymphatic vessel, without sinuses or germinal centers. The second type presents one germinal center and many erythrocytes, scattered at the periphery. The third and the largest stage of LA development is a proper MLN, presenting branching sinuses and many germinal centers embedded into the dense lymphoid tissue [125]. The majority of germinal centers are located on the periphery, but a few of them can be seen at the center of the LA. MLNs have mainly been studied in domestic chickens and very limited information covering other species of fowl is available, except for ducks, where proper lymph nodes resembling (in structure) the mammalian ones have been described [126]. The first histological description of MLNs in domestic fowl was published by Biggs in 1957 [127]. Twenty years later, a detailed morphological description of MLNs was published by McCorkle [128]. Among all MLNs, femoral lymph nodes (FLNs) are the structures that respond to foot-pad injections of antigens like sheep red blood cells (SRBCs) or phytohemagglutinin [129]. FLNs are barely perceptible in physiological conditions. However, following an injection of SRBCs into the foot pad, chickens have been shown to develop more plaque-forming cells in the FLNs than in the spleen, with the colonization of approximately 38% of T cells and 53% of B cells [125]. Foot-pad sensitizations are used in poultry to evaluate immunological profiles, like delayed-type hypersensitivity (DTH) reactions [129,130].

## 3. Conclusions

Birds, more specifically chickens, represent a great model for immune-based studies for both human and veterinary medicine. Humans and birds are indeed often challenged with similar classes of pathogens, against which they appear to build comparable immune responses [3]. This level of conservation among species represents the key for the resolution of major challenges, like the development of suitable alternatives to antibiotics in poultry production and the design of more effective vaccines. The knowledge of poultry immunology is subject to constant development. Therefore, a review summarizing the state of the art surrounding this relevant topic is of great importance for the design of future studies in terms of designing more tailored protocols and implementing improved ethical rules for animal experimentation.

## Figures and Tables

**Table 1:** Immunomodulatory properties of phytobiotics.

Plant-Derived Substances	Major Effects	References
*Aloe vera* extract	Anticoccidial properties and enhancement of growth performance	[86]
*Curcuma longa*, *Scutellaria baicalensis, Spirulina platensis*	Anti-inflammatory effect	[87,88]
Aniseed extract	Positive influence on immune responses, lipid profile, and overall animal performance	[89]
Garlic powder and *Satureja khuzestanica* essential oil	Performance improvement, reduction in serum concentrations of lipid profiles, prebiotic effect, morphological changes (increased villus length to crypt depth and villus area)	[90]
*Ficus religiosa* L. extracts	Immunomodulatory effect and protective properties against coccidiosis	[91]
*Melissa officinalis*	Beneficial effects on the redox balance and improved performance during the growth phase	[92]
Thyme extract	Improvements in growth performance, carcass traits, blood serum parameters, immune responses, and ileal microflora	[93]
Fruit-derived phenolic compounds	Antimicrobial, antioxidant, immunostimulatory, and growth-enhancing effects	[94]
Cinnamon oil	Immunostimulatory, antimicrobial, prebiotic, and growth-enhancing effects	[95]
*Coriandrum sativum* and *Cichorium intybus* extracts	Positive effects on growth performance, carcass characteristics, liver function, serum lipid profile, and antioxidant status	[96]
Salvia and lavender powders	Immunostimulatory and growth-enhancing effects	[97]

## Data Availability

No new data were created or analyzed in this study. Data sharing is not applicable to this article.
